# The Interactive Effects of Cognition on Coping Styles among Chinese during the COVID-19 Pandemic

**DOI:** 10.3390/ijerph18063148

**Published:** 2021-03-18

**Authors:** Zemin Cai, Shukai Zheng, Yanhong Huang, William W. Au, Zhaolong Qiu, Kusheng Wu

**Affiliations:** 1 Department of Preventive Medicine, Shantou University Medical College, Shantou 515041, China; 19zmcai@stu.edu.cn (Z.C.); 15skzheng@stu.edu.cn (S.Z.); 18zlqiu@stu.edu.cn (Z.Q.); 2 Mental Health Center of Shantou University, North Taishan Road, Shantou 515065, China; 12yhhuang@stu.edu.cn; 3 University of Medicine, Pharmacy, Science and Techonology, 540142 Tirgu Mures, Romania; wau@stu.edu.cn; 4 University of Texas Medical Branch, Galveston, TX 77550, USA

**Keywords:** Coronavirus Disease 2019, cognition, stress source, coping style

## Abstract

Background: The outbreak of Coronavirus Disease 2019 (COVID-19) has seriously affected people’s life. The main aim of our investigation was to determine the interactive effects of disease awareness on coping style among Chinese residents during the COVID-19 pandemic. Methods: A total of 616 Chinese residents from 28 provinces were recruited to participate in this investigation. A questionnaire was used to collect demographic characteristics, cognition of COVID-19, and disease-related stress sources. Coping styles were assessed via the Simplified Coping Style Questionnaire (SCSQ). Results: The survey showed that the main source of information on COVID-19 was different in relation to gender, age, educational level, and occupation (*p* < 0.001). People’s knowledge of the disease, preventive measures, and stress factors were different in relation to demographic characteristics (*p* < 0.001). Compared with the baseline values, the scores of positive coping and negative coping based on SCSQ in relation to gender, age, educational level, and occupation were statistically significant (*p* < 0.001, except for participants older than 60 years). Different educational levels corresponded to statistical significant differences in positive coping (*p* = 0.004) but not in negative coping. Conclusions: During the pandemic, people with different characteristics had different levels of preventive measures’ awareness, which influenced their coping styles. Therefore, during public health emergencies, knowledge of prevention and control measures should be efficiently provided to allow more effective coping styles.

## 1. Introduction

In December 2019, Coronavirus Disease 2019 (COVID-19) was reported and became an outbreak in Wuhan, the capital city of Hubei Province, China [1,2]. The disease is due to infection with severe acute respiratory syndrome coronavirus 2 (SARS-CoV-2) [3] and it rapidly spread throughout China. On 30 January 2020, the WHO declared the COVID-19 a global public health emergency [4].

During epidemics of infectious diseases, several key preventive measures must be implemented, such as eliminating the source of infection, cutting off the route of transmission, and protecting vulnerable people [5]. Among these measures and at the outbreak stage of COVID-19, however, the infective source could not be easily eliminated. COVID-19 was verified to spread from person to person via aerosols and contact transmissions [6,7]. This information was used to implement protocols which would cut off transmission and would protect vulnerable populations. Even with preventive and therapeutic guidelines for public health emergencies, the general public usually lack medical knowledge and obtain information through various channels, tending to use personal evaluations to adopt the guidelines. Thus, understanding of COVID-19 by the general public plays a significant role during the pandemic.

A public health emergency is a negative stress source, with characteristics of suddenness, infectivity, and extensiveness [8]. Under these circumstances, the understanding of the disease has a relevant role in individuals’ psychological adjustment. General public’s knowledge of COVID-19 for precautionary measures will affect the initial psychological responses and coping styles during the pandemic. Coping is an individual’s cognition and behavioral effort to reduce stress and the emotional response when dealing with an incident that oversteps one’s abilities to manage adversities [9]. The coping style is a mediating variable between stress source and stress response [10,11]. A study [12] reported that individuals with a positive coping strategy usually had a fighting spirit and a better emotional expression performance, which was considered to indicate good psychological adjustment ability, leading to lower anxiety. Coping styles may also influence anxiety through individuals’ normal or pathological changes of biological parameters [13]. A study [14] confirmed that coping resources, especially social support improving mental health, may exert beneficial effects at least in part by reducing the physiological toll of stress. Therefore, people with an appropriate cognitive and coping style would overcome difficulties associated with the COVID-19 pandemic better than those lacking sufficient coping abilities. However, this topic has not been adequately investigated, especially in China, and information on it would be highly useful for the management of future health emergencies. Therefore, this study was conducted to investigate how people use their disease knowledge and coping styles to choose preventive measures and control COVID-19.

In our study, Chinese residents were surveyed to determine their main source of information on COVID-19, their knowledge of prevention and control measures for COVID-19, the stress factors mainly affecting them, and their coping styles during the outbreak of COVID-19. The aim of this study was to explore the influence of awareness of COVID-19 on coping styles among Chinese residents and provide a scientific basis for carrying out more accurate prevention and control strategies.

## 2. Materials and Methods

### 2.1. Participants and Data Collection

This cross-sectional study was performed via an online investigation using the snowball sampling techniques. Wenjuanxing (http://www.wjx.cn (accessed on 1 February 2020), Changsha Ranxing Information Technology Co., LTD, Changsha, China) was used to collect the sample. The sample size was calculated according to the formula: N = [Max (dimensions) × 10] × [l + 20%]. Chinese residents from 28 provinces were invited to participate in this investigation from 5 February to 25 February 2020. The study was approved by the human ethics committee of the Mental Health Center of Shantou University.

### 2.2. Questionnaire

**Figure 1:**
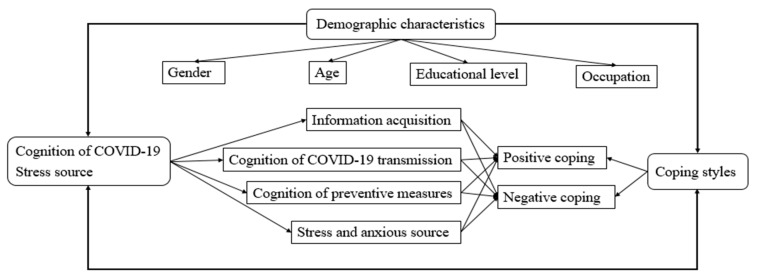
Path analysis of the hypothesized interactive model.

The questionnaire consisted of three parts: (1) General demographic data, including gender, age, educational level, and occupation; (2) Awareness of COVID-19 and stress sources, including the main ways of information acquisition, cognition of COVID-19 transmission routes, cognition of preventive measures, and stress and anxiety factors; (3) Simplified Coping Style Questionnaire, to identify positive and negative coping styles. Path analysis of the hypothesized interactive model was established to evaluate associations between cognition of COVID-19 and coping style (Figure 1).

The Simplified Coping Style Questionnaire (SCSQ) was compiled by Professor Xie [15], based on the cognition theory of coping styles at home and abroad and combined with the characteristics of the Chinese population. The collected data were evaluated for their reliability, and the Cronbach’s α of the SCSQ was 0.90, wherein the Cronbach’s α of the positive coping style was 0.89 and that of the negative coping style was 0.78. The ranking score of each SCSQ item was categorized as follows: not adopted (0), occasionally (1), sometimes (2), usually (3), which would measure the adoption of a more positive coping style in relation to each the domain. The positive coping style subscale consisted of twelve items assessing positive coping characteristics, while the negative coping style subscale involved eight items assessing negative coping characteristics.

### 2.3. Statistical Analysis

Statistical analyses were performed with IBM SPSS Statistics 26.0 (IBM Corp., Armonk, NY, USA). Descriptive statistical analysis was used to describe the characteristics of the participants. Categorical variables were represented as percentages, and quantitative variables were denoted as mean ± standard deviation for continuous data. Chi-square tests were used to compare the data for different categorical variables. Significant differences of continuous variables were assessed by the analysis of variance. All tests were two-sided, and *p* < 0.05 was considered statistically significant.

## 3. Results

### 3.1. Demographic Characteristics of the Participants

A total of 616 participants, 223 males and 393 females, completed the online questionnaire. Most of the participants’ ages ranged from 19 to 59 years (only 3% were 60 years old). More than half of them (71.6%) had an education above the undergraduate level. About 35.9% of the participants had a medical background (Table 1).

### 3.2. The Association between Cognition of COVID-19 and Demographic Characteristics

Path analysis of the hypothesized interactive model between cognition and coping style was established, as shown in Figure 1. The main way the participants acquired information on COVID-19 was via official news and broadcasts, chat software, timely messages from COVID-19-dedicated APPs, and Internet searching. These data showed significant differences depending on gender, age, educational level, and occupation (*p* < 0.05) (Table 2). In particular, statistical significance depending on age and educational level (*p* < 0.05) was reached regarding people’s awareness of COVID-19, aerosol transmission of the disease, contact transmission, fecal or oral transmission, and potential transmission through household items, as shown in Table 3. About the knowledge of preventive measures, such as wearing a mask, avoiding gathering together, washing hands and disinfecting furniture, doing exercise and keeping healthy living habits, the data showed statistical significance depending on gender, age, educational level, and occupation (*p* < 0.05) (Table 4). In regard to the stress and anxious sources, such as limitations to going outside, impact on one’s original schedule, poor awareness of prevention measures in families, impossibility to reunite with one’s family, uncontrolled information and rumors on the disease, and suspected cases around one’s residence, the data showed statistical significance depending on age, educational level, and occupation (*p* < 0.05) (Table 5).

### 3.3. Association between SCSQ and Demographic Characteristics

Univariate analysis of variance was used to analyze gender, age, educational level, and occupation among the participants. Compared with the baseline values, positive and negative coping depending on gender, age, educational level and occupation were statistically significant (*p* < 0.001, except for age ≥ 60 years). When comparing the coping style scores of males and females, we did not find statistical significant differences (*p* > 0.05). Different age groups showed statistically significant differences in both positive coping (*p* < 0.05) and negative coping (*p* < 0.001). People with different educational levels showed statistically significant differences regarding positive coping (*p* < 0.05) but not negative coping (*p* > 0.05). Undergraduates, postgraduates, or above were more likely to get higher scores. When comparing people with or without a medical background, statistically significant differences were not found in positive coping (*p* > 0.05); however, they were found in negative coping (*p* < 0.001) (Table 1).

## 4. Discussion

Our study was conducted to determine how people use their knowledge of the disease and coping styles to deal with the COVID-19 pandemic in China. The results showed that people received information about COVID-19 by multiple media, such as official news and broadcasts, chat software, timely messages from dedicated APPs, and Internet searching. Totally, the Internet was the primary information channel for the Chinese during the initial stage of the COVID-19 pandemic. Internet access was so diversified in terms of visited websites that it might have been difficult to distinguish between real news and rumors [16,17]. Searching information on the Internet has become rife, and even more common during the COVID-19 outbreak. Much of the online information, especially rumors, may cause anxieties and worries [18,19]. Therefore, it is necessary to make full use of official media, refute rumors, and invest more in health education and public health services [20,21].

Most of the surveyed people thought that COVID-19 transmission was via aerosols, direct contact, and fecal/oral routes [22]; they also showed cognition of preventive measures including wearing a mask, avoiding gathering, keeping healthy habits, and so on. These results showed that the surveyed people generally understood the danger and the risks posed by COVID-19 and demonstrated a strong sense of self-protection. During the pandemic, education authorities and enterprises need to develop web-based applications to deliver teaching activities or work tasks [23]. Health authorities could consider providing online or smartphone-based health education, such as on COVID-19 transmission and preventive measures, to reduce risk of virus transmission in face-to-face meetings [24,25]. Government and health authorities need to provide accurate health information during the pandemic to reduce the impact of rumors [26].

The outbreak of COVID-19 is wreaking havoc worldwide, and the pandemic has entered a dangerous new phase [27]. This immediate spread of the pandemic causes anxiety and stress [28]. Various studies [29,30] showed that stress can cause a variety of somatic symptoms as well as mental illness. Psychological stress appears when individuals perceive the impact of the external environment on their emotions and social functions [31]. It can cause adaptive or adverse reactions, are influenced by individual personality characteristics and coping styles [32]. A study [33] reported that ways of coping in stressful conditions do not operate on adjustment in isolation, but on psychosocial parameters in relation to adaptive outcomes. The psychosocial parameters include the characteristics of the stressor, the social context, dispositional attributes, and cognitive appraisals. A public health emergency (COVID-19) is one of the influencing factors of psychosocial parameters. Coping mainly involve changing the evaluation and cognition of the stressful event and adjusting the physical and emotional responses, thus controlling the influence of stressful event [34].

In this study, the effects of information acquisition and cognition of preventive measures for COVID-19 on coping were of statistical significance in different age groups, and the scores of coping styles in different age groups were lower than the baseline scores, which indicates that the public health emergency affected people’s awareness of the disease and coping styles. The positive coping scores of people aged 60 years were higher than those of other age groups, which might be related to the rich social experience and stable psychological qualities of older people [35]. People aged between 19 and 35 years and between 36 and 59 years got similar scores, but still lower than the baseline ones. These age groups included college students and workers in various industries, which indicates the widespread impact of the pandemic on people, especially on those representing the main working force of the society [36]. It is well known that different age groups in different life phases would show variations in response to different types of stressors [37]. For example, young adults would most likely encounter school-related stressors, middle-aged adults work-related stressors, and older adults health-related stressors [38,39]. Our data show that during the pandemic of COVID-19, people of all age groups perceived fear and felt their health threatened and that they adopted different coping styles.

The effects of information acquisition and cognition of preventive measures for COVID-19 on coping were statistically significant, depending on educational levels and the corresponding coping style scores were lower than the baseline ones, with the adoption of a positive coping style having statistical significance. People with undergraduate and postgraduate or above education obtained higher scores than other groups in relation to positive coping, which indicates that people with high educational levels had better knowledge of COVID-19 and Please check if the original meaning is retained. Indeed, their acquired habit of reasoning and logical managing ability would allow them to better cope with emergencies than people with lower educational levels [40]. In addition, our data are consistent with the assumption that a high educational level is a predictive factor for the incidence of non-communicable diseases, with limited mediating effects from socioeconomic status and healthy behavior [41]. A high educational level was reported to improve COVID-19 knowledge among Chinese and to be associated with having a positive attitude and adopting appropriate practices [42]. Intraindividual factors, including coping resources and cognitive appraisals, also affect coping processes. In addition to their role as mediators, coping processes also can interact with contextual and individual parameters in their contribution to adjustment. Newer models for conceptualizing the links among stressful life experiences, coping processes, and mental health outcomes also recognize their potentially reciprocal relations [33], which is consistent with the path analysis of the hypothesized interactive model of our study.

During the pandemic of COVID-19, the scores for positive and negative coping styles of people without a medical background were higher than those of medical workers, which may be link to poor preventive measures’ awareness and lack of professional knowledge to interpret scientific information. At the same time, medical workers were under relatively high stress in a high-risk environment, and the positive coping score of the people without a medical background was higher than that of medical workers, though the difference was not statistically significant. People with a medical background obtained lower scores in negative coping, which is consistent with their professional knowledge and occupational training [43]. Their expertise certainly allowed them to acquire a higher degree of comfort and control over the situations created by the pandemic. In addition, this group would continuously receive updated guidelines [44] and a rapid supply of medical protective items, which allowed them to maintain a positive coping attitude during the COVID-19 pandemic. Therefore, the government and institutions should provide targeted mental and cognitive guidance and carry out behavioral interventions, especially for medical workers during the pandemic, to properly release the pressure they experience.

Limitations and strengths of this study. The present study has several limitations. First, earlier life/stress events and cognition activities were not considered in our study. Second, this study only reflected people’s awareness of COVID-19 and coping style in a phase of the COVID-19 pandemic, though a longitudinal approach might help perform a dynamic observation of these phenomena. Third, detailed random sampling and on-site investigation were difficult to carry out during the pandemic of COVID-19. Despite these limitations, interactive effects were established to evaluate the association between COVID-19 awareness and coping styles of Chinese residents in the early time of the COVID-19 pandemic. In the future, it may be interesting to design a study to investigate the effect of early life events on individuals’ COVID-19 awareness and coping style. A study with dynamic observation of disease cognition and considering behavioral coping interventions should be performed to collect more epidemiological data.

## 5. Conclusions

During the pandemic, people with different individual characteristics showed different levels of COVID-19 preventive measures’ awareness, which influenced their coping styles. Most people adopted a positive coping style, but the coping scores were still below the baseline scores. During public health emergencies, the knowledge of preventive and control measures should be strengthened so to scientifically guide the population to adopting a correct coping style.

## Figures and Tables

**Table 1:** Comparison of the Simplified Coping Style Questionnaire (SCSQ) scores (mean ± SD) based on demographic characteristics.

Variables	*n*	Positive Coping (norm: 1.78 ± 0.52)	Negative Coping (norm: 1.59 ± 0.66)
Gender			
Male	223	1.39 ± 0.06 ***	0.96 ± 0.81 ***
Female	393	1.49 ± 0.86 ***	1.03 ± 0.70 ***
*p*-value		0.166	0.259
Age (years)			
0–18	55	1.04 ± 0.81 ***	0.74 ± 0.72 ***
19–35	356	1.47 ± 0.88 ***	1.00 ± 0.72 ***
36–59	188	1.46 ± 0.88 ***	1.00 ± 0.73 ***
≥60	17	1.69 ± 0.49	1.55 ± 0.19
*p*-value		0.003	<0.001
Educational level			
Secondary school or less	57	1.48 ± 0.99 ***	1.08 ± 0.79 ***
Junior college	118	1.19 ± 0.85 ***	0.87 ± 0.73 ***
Undergraduate	280	1.52 ± 0.89 ***	1.06 ± 0.74 ***
Postgraduate or above	161	1.53 ± 0.86 ***	0.98 ± 0.72 ***
*p*-value		0.004	0.091
Occupation			
With medical background	221	1.42 ± 0.91 ***	0.86 ± 0.72 ***
Without medical background	395	1.47 ± 0.88 ***	1.09 ± 0.74 ***
*p*-value		0.517	<0.001

*** Compared with norm, *p* < 0.001.

**Table 2:** Main ways of acquisition of information on coronavirus disease 2019 (COVID-19) based on demographic characteristics.

Variables	Official News and Broadcasts	Short Message Services	Chat Software	Timely Message of APP	Video Clips of APP	Internet Searching	Others	*p*-Value
Gender								0.039
Males	193 (86.6)	97 (43.5)	138 (61.9)	158 (70.9)	41 (18.4)	110 (49.3)	7 (3.1)	
Females	333 (84.7)	213 (54.2	255 (64.9)	274 (69.7)	92 (23.4)	166 (42.2)	23 (5.9)	
Age (years)								<0.001
0–18	41 (74.6)	37 (67.3)	37 (67.3)	34 (61.8)	27 (49.1)	23 (41.8)	4 (7.3)	
19–35	311 (87.4)	181 (50.8)	210 (59.0)	272 (76.4)	76 (21.4)	155 (43.5)	17 (4.8)	
36–59	157 (83.5)	88 (83.5)	130 (83.5)	116 (83.5)	30 (83.5)	93 (83.5)	9 (83.5)	
≥60	17 (100.0)	4 (23.5)	16 (58.8)	10 (58.8)	0 (0.0)	5 (29.4)	0 (0.0)	
Educational level								<0.001
Secondary school or less	48 (84.2)	23 (40.4)	36 (63.2)	28 (49.1)	10 (17.5)	22 (38.6)	4 (7.0)	
Junior college	98 (83.1)	75 (63.6)	76 (64.4)	70 (59.3)	46 (39.0)	43 (36.4)	7 (5.9)	
Undergraduate	248 (88.6)	141 (50.4)	177 (63.2)	207 (73.9)	52 (18.6)	131 (46.8)	16 (5.7)	
Postgraduate or above	132 (82.0)	71 (44.1)	104 (64.6)	127 (79.0)	25 (15.5)	80 (49.7)	3 (1.9)	
Occupation								0.002
With medical background	198 (89.6)	111 (50.2)	123 (55.7)	155 (70.1)	35 (15.8)	102 (46.2)	8 (3.6)	
Without medical background	328 (83.0)	199 (50.4)	270 (68.4)	277 (70.1)	98 (24.8)	174 (46.2)	22 (5.6)	

**Table 3:** Cognition of COVID-19 transmission based on demographic characteristics.

Variables	Droplet Transmission	Contact Transmission	Fecal/Oral Transmission	Household Articles Transmission	Others	*p*-Value
Gender						0.165
Males	223 (100.0)	196 (87.9)	182 (81.6)	156 (70.0)	15 (6.7)	
Females	390 (99.2)	355 (90.3)	290 (73.8)	266 (67.7)	28 (7.1)	
Age (years)						
0–18	54 (98.2)	48 (87.3)	36 (65.5)	35 (63.6)	1 (1.8)	
19–35	355 (99.7)	310 (87.1)	288 (80.9)	238 (66.9)	20 (5.6)	<0.001
36–59	187 (99.5)	176 (93.6)	132 (70.2)	138 (73.4)	21 (11.2)	
≥60	17 (100.0)	17 (100.0)	16 (94.1)	11 (64.7)	1 (5.9)	
Educational level						
Secondary school or less	54 (94.7)	47 (82.5)	44 (77.2)	37 (64.9)	5 (8.8)	
Junior college	118 (100.0)	104 (88.1)	87 (73.7)	82 (69.5)	6 (5.1)	<0.001
Undergraduate	280 (100.0)	246 (87.9)	215 (76.8)	196 (70.0)	21 (7.5)	
Postgraduate or above	161 (100.0)	154 (95.7)	126 (78.3)	107 (66.5)	11 (6.8)	
Occupation						0.07
With medical background	221 (100.0)	200 (90.5)	171 (77.4)	138 (62.4)	11 (4.98)	
Without medical background	392 (99.2)	351 (88.9)	301 (76.2)	284 (71.9)	32 (8.1)	

**Table 4:** Cognition of preventive measures based on demographic characteristics.

Variables	Wear a Mask	Avoid Gathering Together	Wash Hands and Disinfect Furniture	Exercise	Raise the Room Temperature	Healthy Living Habits	Vinegar Vapors	*p*-Value
Gender								0.003
Males	223 (100.0)	217 (97.3)	213 (95.5)	195 (87.4)	35 (15.7)	204 (91.5)	18 (8.1)	
Females	390 (99.2)	390 (99.2)	392 (99.8)	341 (86.8)	68 (17.3)	349 (88.8)	35 (8.9)	
Age (years)								
0–18	55 (100.0)	55 (100.0)	55 (100.0)	42 (76.3)	15 (27.3)	40 (72.7)	10 (18.2)	
19–35	353 (99.2)	349 (98.0)	352 (98.9)	306 (86.0)	65 (18.3)	326 (91.6)	29 (8.2)	<0.001
36–59	188 (100.0)	186 (98.9)	182 (96.8)	171 (91.0)	22 (11.7)	171 (91.0)	14 (7.5)	
≥60	17 (100.0)	17 (100.0)	16 (94.1)	17 (100.0)	1 (5.9)	16 (94.1)	0 (0.0)	
Educational level								
Secondary school or less	57 (100.0)	55 (96.5)	54 (94.7)	43 (75.4)	10 (17.5)	46 (80.7)	1 (1.8)	
Junior college	118 (100.0)	115 (97.5)	115 (97.5)	95 (80.5)	21 (17.8)	97 (82.2)	17 (14.4)	<0.001
Undergraduate	278 (99.3)	278 (99.3)	279 (99.6)	251 (89.6)	54 (19.3)	259 (92.5)	26 (9.3)	
Postgraduate or above	160 (99.4)	159 (98.8)	157 (97.5)	147 (91.3)	18 (11.2)	151 (93.8)	9 (5.6)	
Occupation								0.050
With medical background	218 (98.6)	216 (97.7)	217 (98.2)	189 (85.5)	33 (14.9)	204 (92.3)	13 (5.9)	
Without medical background	395 (100.0)	391 (99.0)	388 (98.2)	347 (87.9)	70 (17.7)	349 (88.4)	40 (10.1)	

**Table 5:** Stress and anxious sources based on demographic characteristics.

Variables	Limited Going Outside	Going to Work Early	Impact on the Original Schedule	Poor Preventive Measures’ Awareness of Families	Unable to Reunite with Families	Uncontrolled Information and Rumors	Suspected Cases around One’s Residence	Others	*p*-Value
Gender									0.493
Males	151 (67.7)	33 (14.8)	120 (53.8)	47 (21.1)	45 (20.2)	109 (48.9)	54 (24.2)	14 (6.3)	
Females	265 (67.4)	68 (17.3)	175 (44.5)	88 (22.4)	78 (19.9)	195 (49.6)	78 (19.9)	23 (5.9)	
Age (years)									<0.001
0–18	44 (80.0)	2 (3.6)	20 (36.4)	6 (10.9)	5 (9.1)	14 (25.5)	5 (9.1)	3 (5.5)	
19–35	240 (67.4)	65 (18.3)	170 (47.8)	109 (30.6)	71 (19.9)	195 (54.8)	88 (24.7)	19 (5.3)	
36–59	115 (61.2)	34 (18.1)	92 (48.9)	18 (9.6)	46 (24.5)	86 (45.7)	38 (20.2)	15 (8.0)	
≥60	17 (100.0)	0 (0.0)	13 (76.5)	2 (11.8)	1 (5.9)	9 (52.9)	1 (5.9)	0 (0.0)	
Educational level									<0.001
Secondary school or less	43 (75.4)	4 (7.0)	26 (45.6)	6 (10.5)	4 (7.0)	17 (29.8)	5 (8.8)	3 (5.3)	
Junior college	86 (72.9)	8 (6.8)	40 (33.9)	18 (15.3)	21 (17.8)	48 (40.7)	19 (16.1)	8 (6.8)	
Undergraduate	186 (66.4)	53 (18.9)	134 (47.9)	79 (28.2)	59 (21.7)	153 (54.6)	73 (26.1)	19 (6.8)	
Postgraduate or above	101 (62.7)	36 (22.4)	95 (59.0)	32 (19.9)	39 (24.2)	86 (53.4)	35 (21.7)	7 (4.4)	
Occupation									<0.001
With medical background	127 (57.5)	49 (22.2)	106 (48.0)	59 (26.7)	51 (23.1)	122 (55.2)	55 (24.9)	10 (4.5)	
Without medical background	289 (73.2)	52 (13.2)	189 (47.9)	76 (19.2)	72 (18.2)	182 (46.1)	77 (19.5)	27 (6.8)	

## Data Availability

The data presented in this study are available on request from the corresponding author.
